# A novel type I cystatin of parasite origin with atypical legumain-binding domain

**DOI:** 10.1038/s41598-017-17598-2

**Published:** 2017-12-13

**Authors:** Jana Ilgová, Lucie Jedličková, Hana Dvořáková, Michal Benovics, Libor Mikeš, Lubomír Janda, Jiří Vorel, Pavel Roudnický, David Potěšil, Zbyněk Zdráhal, Milan Gelnar, Martin Kašný

**Affiliations:** 1 Department of Botany and Zoology, Faculty of Science, Masaryk University, Brno, 611 37 Czech Republic; 2 Department of Parasitology, Faculty of Science, Charles University, Prague, 128 44 Czech Republic; 3 Central European Institute of Technology, Masaryk University, Brno, 625 00 Czech Republic

## Abstract

Parasite inhibitors of cysteine peptidases are known to influence a vast range of processes linked to a degradation of either the parasites’ own proteins or proteins native to their hosts. We characterise a novel type I cystatin (stefin) found in a sanguinivorous fish parasite *Eudiplozoon nipponicum* (Platyhelminthes: Monogenea). We have identified a transcript of its coding gene in the transcriptome of adult worms. Its amino acid sequence is similar to other stefins except for containing a legumain-binding domain, which is in this type of cystatins rather unusual. As expected, the recombinant form of *E*. *nipponicum* stefin (rEnStef) produced in *Escherichia coli* inhibits clan CA peptidases – cathepsins L and B of the worm – via the standard papain-binding domain. It also blocks haemoglobinolysis by cysteine peptidases in the worm’s excretory-secretory products and soluble extracts. Furthermore, we had confirmed its ability to inhibit clan CD asparaginyl endopeptidase (legumain). The presence of a native EnStef in the excretory-secretory products of adult worms, detected by mass spectrometry, suggests that this protein has an important biological function at the host-parasite interface. We discuss the inhibitor’s possible role in the regulation of blood digestion, modulation of antigen presentation, and in the regeneration of host tissues.

## Introduction

*Eudiplozoon nipponicum* (Goto, 1891) is a blood-feeding oviparous monogenean of East Asian origin which frequently parasitizes the gills of the common carp, *Cyprinus carpio*. As in other haematophagous helminths, adults use the host’s blood as a valuable source of carbohydrates, proteins, and fatty acids for their metabolism, growth, and egg production^1^. Blood uptake and constant attachment to the host’s tissue is related to several complex mechanisms such as continuous blood digestion, intracellular accumulation/release of residual haematin pigment, inflammation of host tissue, and evasion of host defence mechanisms^1,2^. In order to successfully manage these processes, blood-feeders produce a variety of bioactive compounds, which are frequently found in their excretory-secretory products^3,4^. A recent characterisation of *E*. *nipponicum* cysteine and aspartic peptidases (cathepsins L, B and D) has clarified their role in haemoglobin processing^5^. Activity of these peptidases can be regulated by endogenous inhibitors, and cystatins have been shown to possess such a regulatory function^6^. As a result, they can influence many physiological processes related to protein degradation in both the parasites and their hosts (reviewed in^7,8^).

In general, cystatins are competitive, reversible, tight-binding inhibitors of cysteine peptidases, which bind to the same peptidase active site as the protein/peptide substrate^9^. Target enzymes of cystatins belong to C1 (clan CA, papain-like peptidases) and C13 (clan CD, legumain-like peptidase) families^10^. C1 family peptidases, earlier believed to be a kind of lysosomal enzymes, often have also non-endosomal roles, such as protein degradation during digestion. In addition to being involved in protein turnover, they participate in endosomal antigen presentation and in signalling pathways^9^. It has been well established that legumain, also known as asparaginyl endopeptidase, is connected with endopeptidase activity. Its key physiological functions are the mediation of haemoglobin degradation^11^, protein processing for antigen presentation^12^, and proteolytical activation of TLRs^13^. Recent research has also confirmed its ability to act as a carboxypeptidase and peptide ligase^14^.

According to the MEROPS classification^10^, single-domain cystatins with inhibitory capacity are divided into subfamilies I25A and I25B. The former, also known as stefins or type I cystatins, include intracellular inhibitors of peptidase representatives from the C1 peptidase family. The stefin molecule typically consists of ca. 100 amino acids (MW ≈ 11 kDa) and does not contain disulphide bonds. The latter group, i.e. the subfamily I25B inhibitors, also known as cystatins or type II cystatins, includes secreted proteins consisting of ca. 120 amino acids (MW ≈ 14 kDa) whose tertiary structure is stabilised by disulphide bonds. The acquisition of a signal peptide by I25B cystatins was an important evolutionary event responsible for extracellular targeting in this group of inhibitors^15^. Type II cystatins mostly act on C1 family peptidases, although some are able to act upon C13 family peptidases (legumains) through a second independent reactive site^16^.

The production of both stefins and type II cystatins has been described in all groups of parasitic organisms. They play a fundamental role in endogenous processes such as regulation of haemoglobin degradation in schistosome species^17^, heme detoxication in ticks^18,19^, protection of intestinal epithelial lining against inappropriate endogenous proteolysis by cysteine peptidases in *Clonorchis sinensis* ^20^, or regulation of the moulting process in the larvae of *Onchocerca volvulus* ^21^. Stefins and cystatins also contribute to the innate immunity against microorganisms in ectoparasitic arthropods and leeches^18,22^. Additionally, parasite stefins/cystatins target a number of host’s cysteine peptidases, which are essential for both innate and adaptive immunity, antigen presentation, and a regulation of pattern recognition and receptor signalling^7^. It has been proposed that these inhibitors have a significant immunomodulatory and anti-inflammatory effect, since infections caused by the parasites are frequently accompanied by a shift in the host’s cytokine balance from Th1 toward a Th2 response^23^.

Our knowledge of the functional molecules produced by the diverse and cosmopolitan group of monogenean parasites is still incomplete, fragmentary, and based on just a few representatives, such as the *Neobenedenia girellae* serine peptidases^24^, *Neobenedenia melleni* cathepsin L^25^, *Microcotyle sebastis* annexin^26^, and *E*. *nipponicum* cathepsins L/B/D^5^. In order to better understand monogenean peptidase inhibitors, and cystatins in particular, further research is clearly needed. In the current study, we performed a structural and functional characterisation of a novel type I cystatin (stefin) produced by the *E*. *nipponicum*. It is the first molecule of this type observed in monogeneans.

## Results

### The cloning of EnStef and an *in silico* structural and comparative analysis

**Figure 1:**
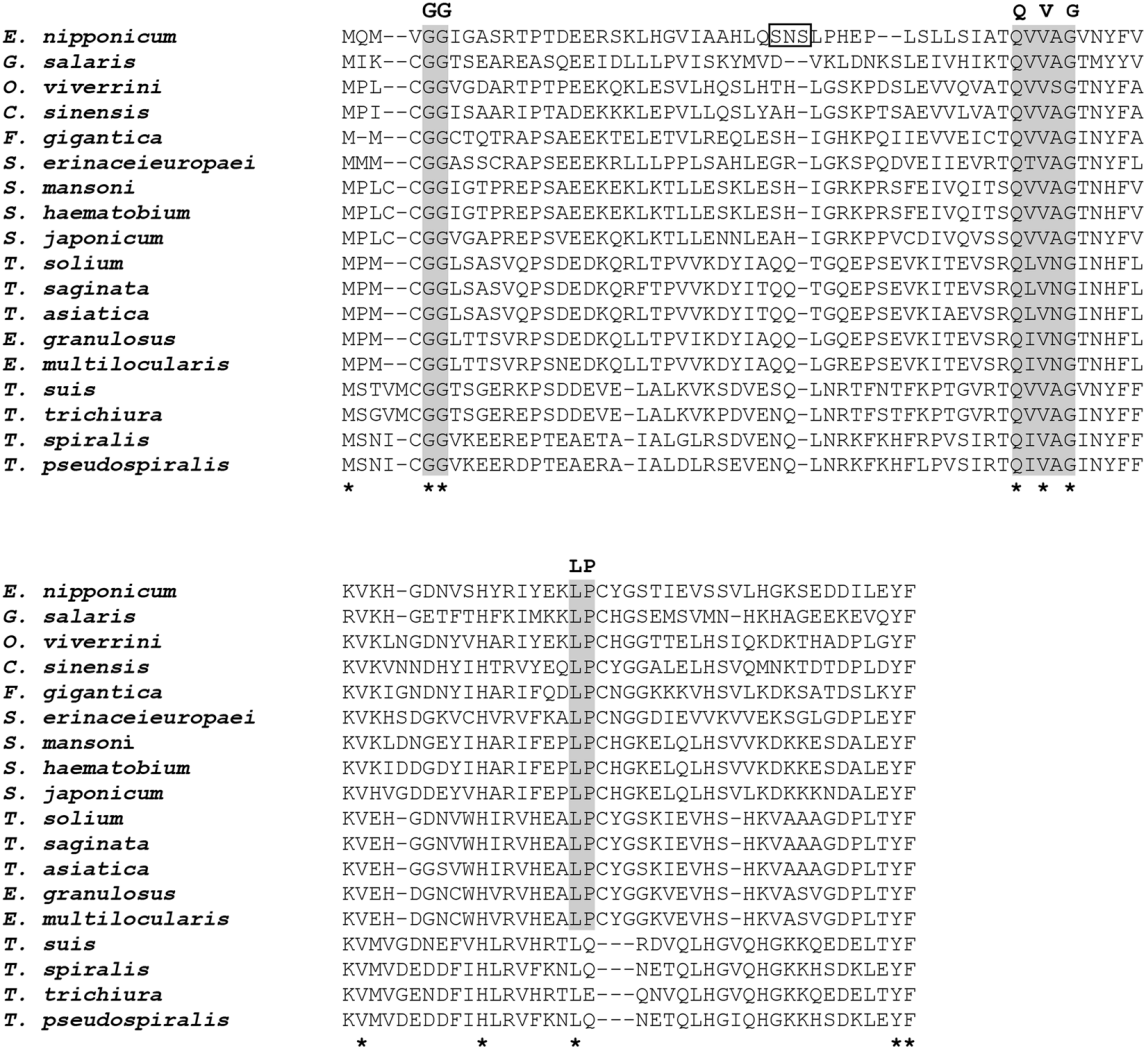
Multiple protein sequence alignment and conserved domains of EnStef and stefin orthologs from selected platyhelminths and nematodes. Alignment demonstrates the position of conserved inhibitory domains. Motifs critical for the binding of papain-like peptidases (G5G6, Q47VVAG51 and L73P74) are highlighted in grey. The legumain-binding domain (S31NS33) is indicated in a box in the EnStef sequence. Consensus residues are marked by an asterisk (the numbering applies to native EnStef).

The *E*. *nipponicum* stefin sequence (297 bp) was retrieved after PCR, cloning, and sequencing using primers designed on the basis of a stefin sequence found in the transcriptomic data (see Additional Information). The absence of a signal peptide was verified by 5′ RACE PCR, followed by a sequencing of the resulting amplicon (341 bp). The 297 bp *E*. *nipponicum* stefin sequence encodes a 98 amino-acid protein with a predicted molecular weight of 10.8 kDa and a pI value of 6.02. The sequence length, as well as absence of both disulphide bonds and a signal peptide, correlates with the general properties of type I cysteine peptidase inhibitors (stefins). The EnStef amino acid sequence has three conserved regions typical of stefins, namely a pair of N-terminal glycins (G5G6), a central Gln-Val-Val-Ala-Gly motif (Q47VVAG51), and a Leu-Pro (L73P74) motif. All these regions are critical for binding the papain-like cysteine peptidases^6^ (Fig. 1). These three highly evolutionarily conserved sites (GG, QxVxG, LP) have been recognised in the amino acid sequence alignment of the EnStef and stefin orthologs found in other platyhelminth species (Fig. 1). Nematode stefins share the first two sequence characteristics (GG, QxVxG) with platyhelminths, but lack prolin in the LP motif. Surprisingly, the EnStef sequence also incorporates a potential legumain-binding motif, Ser-Asn-Ser (S31NS33), previously found only in type II cystatins of parasitic nematodes or in the type II cystatins C, F, and E/M of mammals^16^.

**Figure 2:**
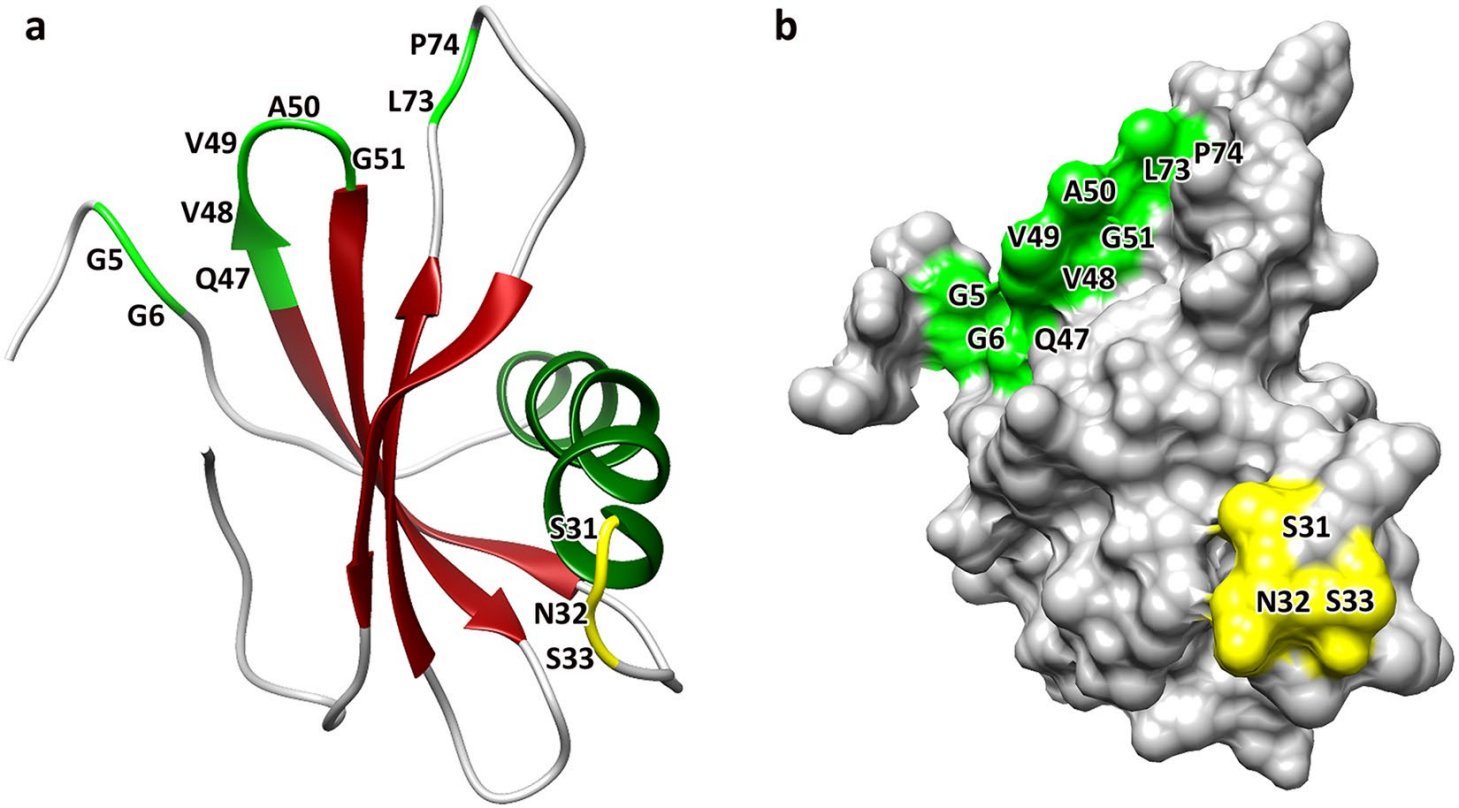
Predicted 3D structure for the *Eudiplozoon nipponicum* stefin. The EnStef 3D structure comprises a four-strand, antiparallel beta-sheet, and a five-turn alpha-helix. (**a**) Motifs critical for binding to papain-like peptidases (G5G6, Q47VVAG51 and L73P74) are highlighted in light green, while the asparaginyl endopeptidase-binding domain formed by S31NS33 residues is highlighted in yellow (the numbering applies to native EnStef). (**b**) Surface structures included in the 3D model.

We have used the Phyre2 web tool^27^ to select two templates for modelling the 3D structure of EnStef based on heuristics in order to maximise confidence, percentage identity, and amino acid alignment coverage. The first template referred to human stefin A (PDB ID: 1NB5)^28^, the second to human stefin B (PDB ID: 1STF)^29^. According to these two templates, the most probable 3D structure for EnStef was built with 98% of residues modelled with over 90% confidence, whereby only two residues were modelled *ab initio*. The theoretical tertiary structure consists of one right-hand-coiled alpha-helix comprising almost five turns and one beta-sheet formed from four antiparallel beta-strands (Fig. 2). Conserved motifs characteristic of stefins which inhibit papain-like peptidases were highlighted in the model. The first (GG) is located after the first four residues from the N-terminus, the other two (QVVAG and LP) in the hairpin loops between the first and second and the third and fourth beta-strands, respectively. The unique domain which binds asparaginyl endopeptidases (legumains) consists of three amino acids (SNS), is located directly after the alpha-helix (Fig. 2).

### Phylogenetic analysis

**Figure 3:**
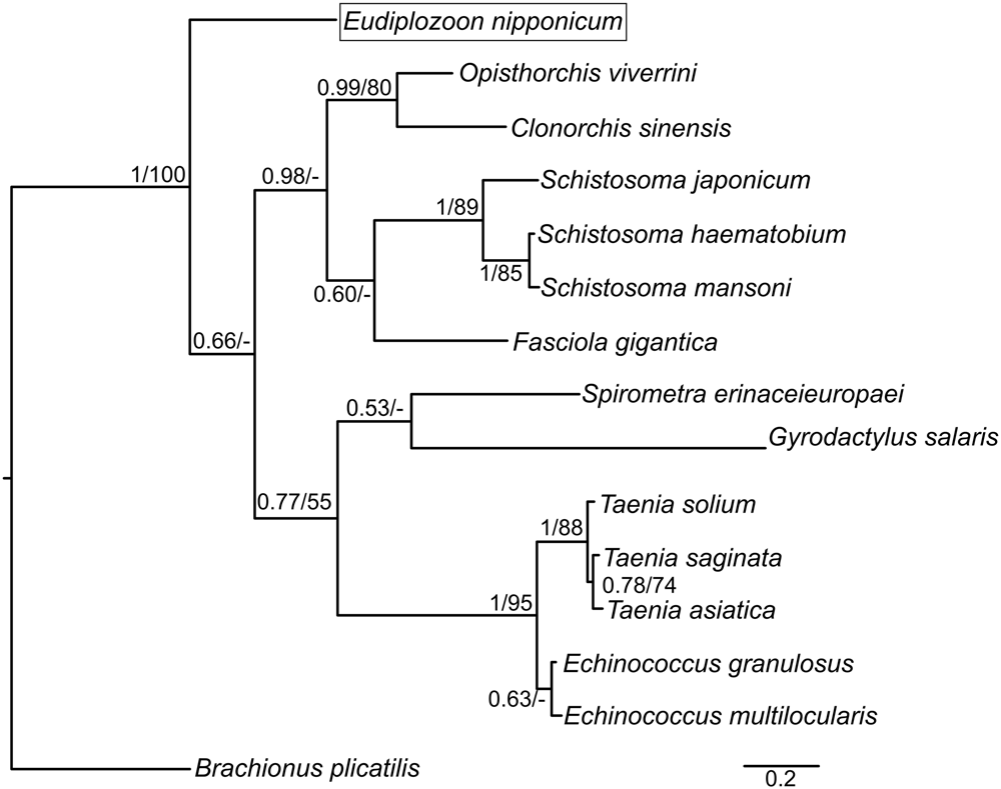
Phylogram of platyhelminth type I cystatins (stefins), based on a Bayesian inference analysis. Values along the branches indicate posterior probabilities and bootstrap values yielded by Bayesian inference and maximum likelihood analysis, respectively (only values above 50 shown). Branch length corresponds to expected amino acid substitutions per site. *Brachionus plicatilis* stefin ortholog was used for rooting the tree.

The final sequence set used for a phylogenetic analysis contained 15 unambiguously aligned protein sequences, each 102 amino acids long (Supplementary Information, S1). Optimal parameters (α = 1.26) and evolution model (LG + G) were calculated using ModelGenerator v. 0.851 and applied during a Bayesian inference and a Maximum likelihood analysis. Both methods provided trees with similar topologies. We therefore present the Bayesian inference tree with posterior probabilities and Maximum likelihood bootstrap values (Fig. 3 **)**.

Phylogenetic analysis confirmed that *E*. *nipponicum* and *Gyrodactylus salaris* monogenean stefin sequences do not form a monophyletic group: the *G*. *salaris* stefin ortholog clustered together with cestode stefins (moderately supported by BI and ML analyses). EnStef forms a sister clade to the stefin orthologs of other platyhelminths. Cestode stefins form a paraphyletic clade, while trematode stefins form a monophyletic group (highly supported by BI analysis and weakly supported by ML analysis).

### Expression and purification of the recombinant EnStef (rEnStef)

**Figure 4:**
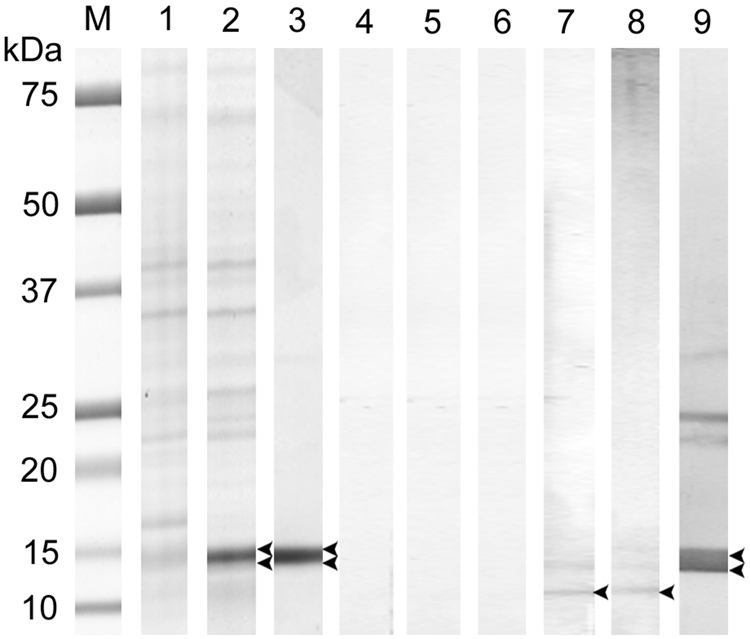
The expression of rEnStef and generation of the anti-rEnStef serum. Columns 1–3: Protein fractions from induced rEnStef expression in *Escherichia coli* on an SDS-PAGE gel stained with Coomassie blue. (M) protein standard, (1) non-induced control, (2) induced soluble fraction, (3) soluble Ni-NTA affinity-purified rEnStef with the N-terminal His-tag and enterokinase sites isolated from pET19b-rEnStef *E*. *coli* transformants. Columns 4–9: Western blot detection of rEnStef and native EnStef in *Eudiplozoon nipponicum* excretory-secretory products and in the soluble worm extract by rabbit serum. Columns 4–6 were incubated with a pre-immune serum and columns 7–9 with the anti-rEnStef serum. Columns 4 and 7 = excretory-secretory products (50 µg), columns 5 and 8 = soluble worm extract (50 µg), columns 6 and 9 = rEnStef (3 µg). Double arrowheads point to the rEnStef (containing N-terminal His-tag and enterokinase site, theoretical MW 13.75 kDa). Single arrowheads indicate the position of a native EnStef (theoretical MW 10.8 kDa).

**Figure 5:**
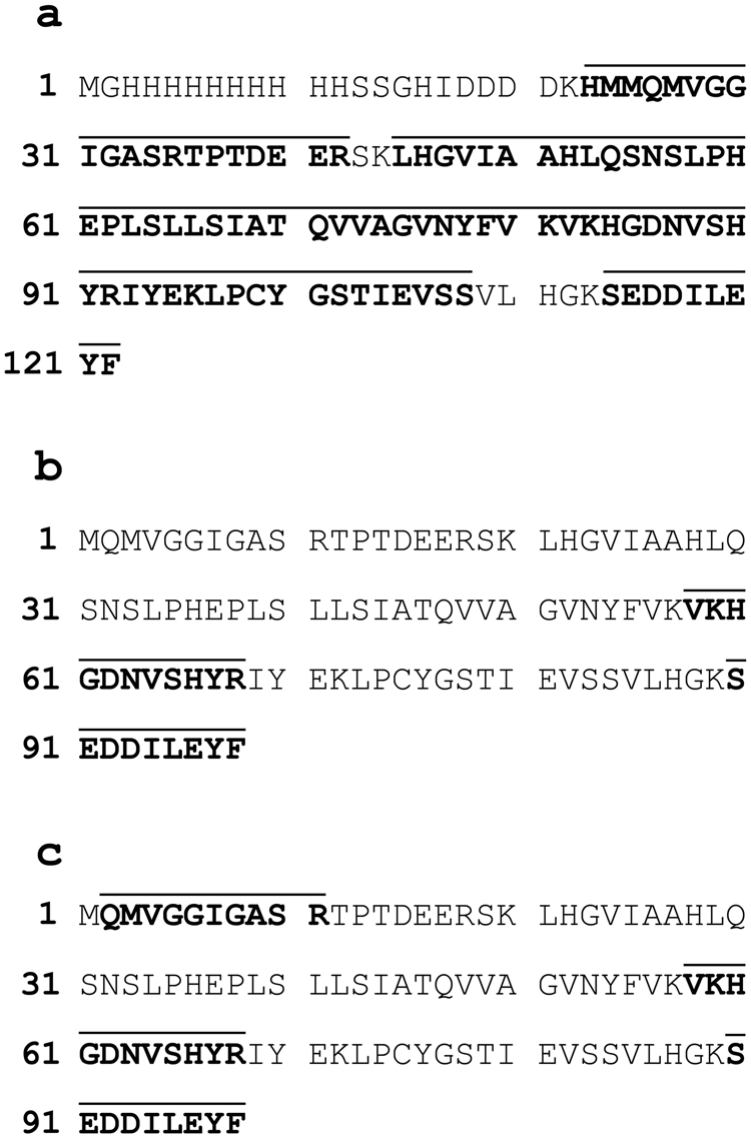
Identification of rEnStef and native EnStef by mass spectrometry. Amino acid residues identified in the tryptic digests of *Eudiplozoon nipponicum* rEnStef, excretory-secretory products, and the soluble worm extract are shown in bold, with total coverage marked by a line above the sequence. (**a**) rEnStef (with His-tag and enterokinase site) from the soluble fraction obtained after a recombinant expression in *Escherichia coli* (11 peptides detected, 76% coverage); (**b**) excretory-secretory products range of MW ca. 10–16 kDa (2 peptides detected, 20% coverage); (**c**) soluble worm extract range of MW ca. 10–16 kDa (3 peptides detected, 31% coverage).

A recombinant *E*. *nipponicum* stefin (rEnStef) was expressed as a His-tagged protein in *E*. *coli* and purified using Ni-NTA affinity chromatography (Fig. 4). The production of rEnStef was verified by mass spectrometry, with 11 peptides covering 76% of the sequence which was identified in a protein band of corresponding size that was separated by SDS-PAGE (Fig. 5a).

### Western blot analysis and detection of EnStef in the excretory-secretory products and soluble worm extract by mass spectrometry

Rabbit anti-rEnStef sera reacted with both His-tagged rEnStef (ca. 14 kDa) and with the native protein in both excretory-secretory products and in soluble worm extracts (ca. 11 kDa) (Fig. 4). Mass spectrometry also confirmed the presence of EnStef in samples separated by the SDS-PAGE (bands analysed between ca. 10–16 kDa). Two stefin peptides (coverage 20%) were identified in the excretory-secretory products (Fig. 5b) and three (coverage 31%) in the soluble worm extract (Fig. 5c).

### Inhibition assays

The inhibitory effect of rEnStef was tested on selected cysteine peptidases from the C1 family, namely cathepsin B and L3, both originating in the *E*. *nipponicum* (EnCB and EnCL3), and on the mouse cathepsin L (MmCL). The asparaginyl endopeptidase from the C13 family of cysteine peptidases originated in the *Ixodes ricinus* tick (IrAe).

**Figure 6:**
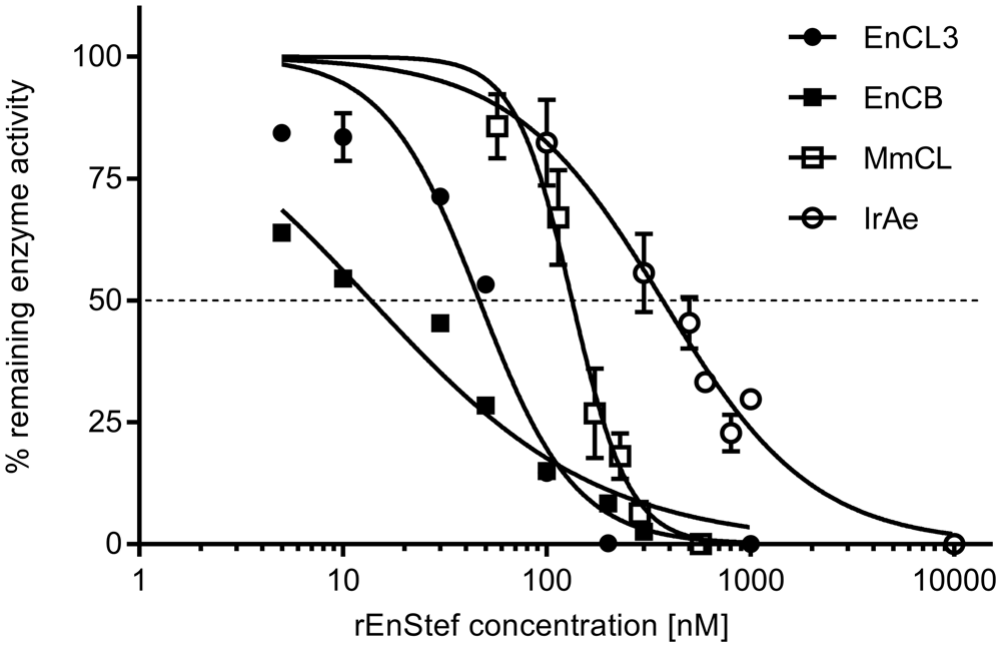
Inhibition assays with rEnStef. Inhibition of *Eudiplozoon nipponicum* cathepsins L3 (EnCL3) and B (EnCB), mouse cathepsin L (MmCL), and *Ixodes ricinus* asparaginyl endopeptidase (IrAE) by rEnStef. The dashed line indicates a 50% inhibition of peptidase activities (IC_50_; values listed in Table 1).

Cathepsins EnCB and EnCL3 were inhibited comparably by EnStef, which exhibited the strongest effect among the tested enzymes. The lowest IC_50_ value was detected for EnCB (13.7 nM), while the inhibition of MmCL was less efficient. In the case of the IrAe, the value of the IC_50_ was by an order of magnitude higher than that observed for the EnCB (Table 1). Inhibition curves obtained for EnCB and IrAe exhibit gentler slopes than those describing the behaviour of EnCL3 and MmCL (Fig. 6).

**Table 1:** IC_50_ values of recombinant stefin of *Eudiplozoon nipponicum* for the inhibition of mouse cathepsin L (MmCL); *Eudiplozoon nipponicum* cathepsins L3 (EnCL3) and B (EnCB); and *Ixodes ricinus* asparaginyl endopeptidase (IrAe).

Peptidase	Peptidase concentration	IC_50_ (confidence intervals) [nM]
MmCL	5 nM	133.1 (122.3–143.5)
EnCL3	10 nM	46.66 (40.17–53.16)
EnCB	10 nM	13.7 (11.45–16.17)
IrAe	10 nM	370.7 (326.1–416.4)

### Inhibition of haemoglobinolysis by rEnStef

**Figure 7:**
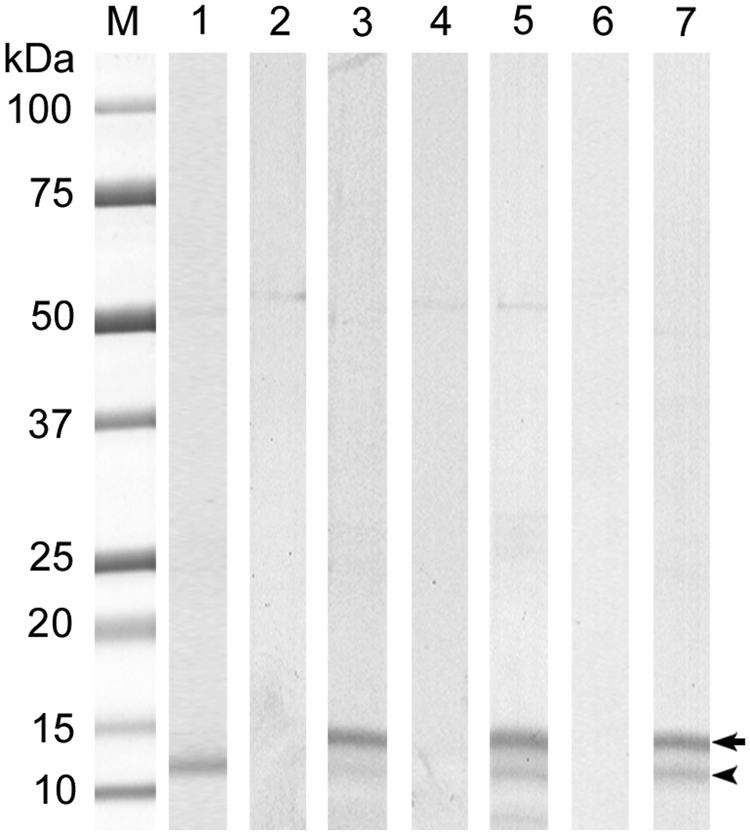
Inhibition of haemoglobin degradation by EnCL3, excretory-secretory products, and soluble worm extract in the presence of rEnStef. SDS-PAGE gel stained with Coomassie brilliant blue R-250. (M) protein standard; (1) haemoglobin; (2) haemoglobin + EnCL3; (3) haemoglobin + EnCL3 + rEnStef; (4) haemoglobin + excretory-secretory products; (5) haemoglobin + excretory-secretory products + rEnStef; (6) haemoglobin + soluble worm extract; (7) haemoglobin + soluble worm extract + rEnStef. The arrow points to rEnStef, while the arrowhead indicates intact haemoglobin. Note the inhibition of haemoglobin degradation caused by rEnStef in columns 3, 5 and 7.

Peptidolytic assays indicated that haemoglobin was efficiently degraded in the presence of *E*. *nipponicum* excretory-secretory products, soluble worm extract, and EnCL3. Haemoglobinolytic activity in all samples was partially inhibited by the rEnStef at pH 5.0 (Fig. 7).

## Discussion

While the morphological and anatomic features of the blood-feeding fish ectoparasite *Eudiplozoon nipponicum* (Monogenea) have been described in considerable detail^30–33^, our understanding of its molecular physiology and host-parasite interactions is still limited. In order to gain a better insight into these issues, we tried to identify and characterise the structural and functional attributes of a type I cysteine peptidase inhibitor (stefin) produced by this ectoparasite. It is well-known that cystatins play an important role in the regulation of a whole range of physiological processes in parasites (including their digestion), and it is possible that they also have a key function in altering the host’s immunity^34^.

The *E*. *nipponicum* cysteine peptidase inhibitor (EnStef) displays the typical length of type I cystatins (98 amino acids) and shares most of its sequential and structural characteristics with other type I cystatins, which are also known under the name stefins (I25A subfamily)^6^. Similarly to other platyhelminth stefin orthologs identified by combined computational approach^35^, EnStef lacks a signal peptide and the PW motif, but include the QxVxG and LP domains.

*In silico* 3D modelling revealed substantial structural similarities between EnStef and human stefin B^29^. Nonetheless, the short beta-strand that precedes the alpha-helix in human stefin was not found in the EnStef structure. This suggests that the EnStef 3D structure contains a four-stranded beta-sheet, rather than a five-stranded beta-sheet such as is found in human stefin A and B.

According to the MEROPS classification^10^, type I cystatins inhibit only papain-like peptidases (clan CA, family C1). On the other hand, several type II cystatins, found in many taxa, have the ability to inhibit not only peptidases of the clan CA family C1, but also legumain (clan CD cysteine peptidase, family C13)^16,36^. This capacity has been attributed to a ‘legumain-binding’ motif formed by three residues, S38ND40/SNS/TND (human cystatin C numbering), and located between the first conserved glycine and the QxVxG domain. Sequence alignments and 3D molecular modelling of human type II cystatins indicate that this motif forms a ‘back-side loop’ located opposite the papain-binding surface. Preserved inhibitory activity of mutant cystatin C variant (N39 substituted for K39) with respect to cathepsin B, together with its inability to inhibit legumain, then highlights the importance of the N39 residue for the inhibition of legumain^16^.

Surprisingly, the legumain-binding domain (S31NS33) was recognised in the EnStef sequence and it provides an efficient inhibition of legumain (asparaginyl endopeptidase) from the tick *I*. *ricinus* (IrAE). Similarly to type II cystatins (cystatin C, cystatin E/M, cystatin F), the legumain-binding motif of EnStef is located between an alpha-helix and the first beta-strand on the side opposite the papain-binding surface. Because it has two non-overlapping inhibitory sites, EnStef is capable of simultaneously blocking the activity of both the papain-like cysteine peptidases and asparaginyl endopeptidases. To the best of our knowledge, type I cystatins (stefins) capable of inhibiting legumains have not yet been described in any scientific report. This is thus the first report of a type I cystatin capable of inhibiting peptidases from the C13 family.

According to the most extensive evolutionary study of the cystatin superfamily^15^, a duplication of ancestral cystatin gene led to the formation of two independent lineages (ancestral stefins and type II cystatins). It is assumed that the formation of a legumain-binding domain found in several type II cystatins must have taken place after this duplication^16^. The presence of a legumain-binding domain in the EnStef sequence and its absence in known stefin orthologs of other species lead us to hypothesise that the formation of this domain in EnStef sequence had been induced by an independent evolutionary event. Further research of stefin orthologs from diplozoid and other polyopisthocotylean species is needed to verify whether this inhibitory domain is found indeed only in *E*. *nipponicum* or also in other related taxa.

A reconstruction of phylogenetic relationships among platyhelminth stefins revealed a position of the EnStef as a sister clade to the stefin orthologs of other Platyhelminth taxa. Cestode and trematode stefin orthologs, meanwhile, form a paraphyletic and monophyletic clade respectively. A stefin ortholog from another monogenean parasite, the *G*. *salaris*, seems to have a closer evolutionary relationship with cestode stefin orthologs than the EnStef, but its position within the clade is only weakly supported by both Bayesian and Maximum likelihood analyses. The presence of a legumain-binding domain (S31NS33) makes the EnStef unique among platyhelminth stefin orthologs.

Cystatins block the catalytic site of cysteine peptidases by competitive inhibition, thereby controlling the proteolytic activity of their targets^37^. Kinetic measurements of rEnStef inhibitory capacity toward cysteine peptidases showed a considerable effect on the activity of recombinant *E*. *nipponicum* cathepsins L3 and B, whereby the inhibition of EnCB was yet somewhat stronger. This is a surprising observation because an efficient inhibition of cathepsin L and a weak inhibition of cathepsin B have been considered a general feature of cystatins^38^. Inhibition of cathepsin B requires a two-step mechanism (a relocation of the ‘occluding loop’ prior to active site binding), whereas papain and several other cysteine cathepsins are inhibited by a one-step reaction^9^. On the other hand, the inability of some stefins to efficiently inhibit particular cathepsins may have led to a functional diversification, which resulted in a varying specificity of stefins produced by a single species. For instance, the digenetic trematode *Fasciola gigantica* secretes two stefins: one is evolutionarily adapted to inhibiting cathepsin B, the crucial peptidase for penetration of newly excysted metacercariae during the initial stages of infection^39^, while the other efficiently inhibits cathepsins S and L, although its blocking of cathepsin B is only very partial^40^. Our finding of a minute difference in the inhibition of EnCL3 and EnCB suggests that EnStef may be adapted to regulating the activity of both.

Although stefins are mainly intracellular inhibitors, they are frequently found in body fluids, such as human urine^41^, or in the excretory-secretory products of several trematodes, such as *F*. *gigantica*, where they either regulate intracellular processes or impact the parasite-host interactions on an extracellular level^39,40^. Interestingly, the EnStef has been identified among the compounds released by the monogenean parasite even though its sequence does not contain a signal peptide. The absence of this signal sequence suggests the existence of a hitherto unknown alternative way of stefin transport from the intracellular environment into the excretory-secretory products. The structural features of EnStef, its presence in excretory-secretory products, and its efficient inhibition of cathepsin L, cathepsin B, and legumain all call for a closer discussion of the possible functions of this novel stefin.

Interestingly, haematophagous monogeneans process host haemoglobin within the intestinal cells. This intracellular localisation of the process is a feature they share with ticks rather than, for instance, with endoparasitic helminths^1,42,43^. Digestion within the lysosomal cycle takes place after a pinocytosis of haemoglobin by the epithelial cells of the gastrodermis^43–45^. The main haemoglobinolytic enzymes in *E*. *nipponicum* are cysteine and aspartic peptidases, which may function within a proteolytic cascade similar to that known from some other parasitic blood feeders^1,5^. The inhibitory effect of the EnStef on *E*. *nipponicum* digestive peptidases thus suggests that they may play role in regulating the digestive process. Analogously, tick cystatins regulate haemoglobin degradation, heme detoxication, and midgut lysosomal activity^18,19,46^.

The ability to inhibit legumain – i.e., the peptidase responsible for trans-activation of some other digestive peptidases in addition to its own haemoglobinolytic activity in schistosomes and other helminths^47,48^ – indicates that the EnStef may be involved in the regulation of the whole proteolytic cascade within lysosomes of the gastrodermis. Although fluorometry failed to detect any activity of aspartic endopeptidases in *E*. *nipponicum* ^5^, a transcript coding for legumain was found in the transcriptome of adult worms (data awaiting publication).

It is believed that parasite cystatins attack the antigen presentation process that is carried out by host immune cells by inhibiting the processing of both the exogenous antigens and the major histocompatibility complex class II-associated invariant chain by lysosomal cysteine peptidases, including cathepsins L and B, and legumains^7^. The properties of EnStef, and its presence in worm excretory-secretory products make it a good candidate for this kind of modulation of fish immunity. Moreover, EnStef could moderate the inflammatory reactions at the gill attachment site by inhibiting cysteine peptidases released by the immune cells. Blood-feeding gill parasites frequently cause hyperplastic responses in the infected tissue which are accompanied by infiltration of inflammatory cells into the affected tissue^49,50^. Absence of such an intense tissue damage during an infestation by diplozoid monogeneans^51^ may indicate an that *E*. *nipponicum* is capable of an elaborate modulation of the host defence mechanisms.

In conclusion, we first characterised a stefin-type inhibitor which in addition to the regular papain-binding motif rather surprisingly contains a legumain-binding domain and thus effectively inhibits asparaginyl endopeptidases. Additionally, it is also the first peptidase inhibitor described in a species belonging to the large group of monogenean parasites. Despite the great economic importance of this issue, fish and their interactions with parasites tend to be rather neglected by biochemical and molecular research. The recombinant molecule we produced provides an opportunity for studying a broad spectrum of proteolysis-dependent biological processes both in the parasite and in the fish host parasitized by blood-feeding monogeneas, including especially the regulation of digestion, immunomodulation, and tissue regeneration.

## Methods

### Parasite material

Adult *E*. *nipponicum* were collected from *C*. *carpio* caught in the littoral zone of the Mušov lowland reservoir (48°53′12″N, 16°34′37″E; Czech Republic). Soluble worm extract and excretory-secretory products were prepared as described previously^5^, whereby the protein concentration was determined using the Quaint-iT^TM^ Protein Assay Kit (Life Technologies) and a SpectraMax i3 fluorometer (Molecular Devices). Samples of the *E*. *nipponicum* tissue for isolating the RNA were immersed in RNAlater^®^ Solution (Roche). All samples were stored in −80 °C.

Ethics statement: All procedures performed in studies involving animals were carried out in accordance with European Directive 2010/63/EU and Czech laws 246/1992 and 359/2012 which regulate research involving animals. All experiments were performed with the legal consent of the Animal Care and Use Committee of Masaryk University and of the Research and Development Section of the Ministry of Education, Youth, and Sports of the Czech Republic.

### *In silico* structural analysis

A full-length *E*. *nipponicum* stefin (cystatin I) sequence (297 bp) was obtained from completely assembled transcripts from *E*. *nipponicum* transcriptome deposited in the NCBI GenBank database (see Additional Information). Protein-coding transcripts served as a nucleotide database and the BLAST + tool v. 2.2.30^52^ (BLASTn, E-value: 10e-5) was employed to carry out a similarity search using platyhelminth stefin query sequences of *Clonorchis sinensis* (ABR68548), *Fasciola gigantica* (ACS35603), and *Opisthorchis viverrini* (KER31316) obtained from GenBank (accession codes are in the parentheses). EnStef parameters were predicted using the ExPASy ProtParam online tool^53^ and SignalP 4.1^54^. Prediction of secondary and tertiary structures was carried out using the Phyre2 online tool set to intensive mode^27^. Subsequent molecular graphics and analyses were performed using UCSF Chimera v. 1.10.2^55^ in combination with Modeller 9.16^56^.

### Phylogenetic analysis

The EnStef amino acid sequence was compared to stefin sequences from other platyhelminths and nematodes using fast Fourier transform in MAFFT^57^. The following GenBank sequences of stefin orthologs (accession codes in the parentheses) were used for the alignment in order to set apart from other taxonomic groups the conserved inhibitory domains of stefins: *O*. *viverrini* (KER31316); *Clonorchis sinensis* (ABR68548); *Fasciola gigantica* (ACS35603); *Schistosoma mansoni* (AAQ16180); *Schistosoma haematobium* (KGB39898); *Taenia solium* (AIM55118); *Spirometra erinaceieuropaei* (AGC74033); *Schistosoma japonicum* (CAX73577); *Taenia saginata* (OCK38197); *Taenia asiatica* (AIM55121); *Echinococcus granulosus* (EUB56763); *Echinococcus multilocularis* (CDI97789); *Gyrodactylus salaris* (KL566595); *Trichinella spiralis* (KY192530); *Trichinella pseudospiralis* (KRX94825); *Trichuris suis* (KFD70667); *Trichuris trichiura* (CDW53641). The *G*. *salaris* GenBank accession number refers to the whole genome shotgun sequence of *G*. *salaris* from which the stefin sequence was mined. The alignment of stefin orthologs of the nematodes and trematodes with highlighted inhibitory domains is shown in Fig. 1. To reconstruct evolutionary relationships among stefin orthologs of platyhelminths, we have selected 14 sequences and a stefin ortholog of a Rotifera representative (*Brachionus plicatilis*). Sequence alignment was checked and trimmed manually to remove gaps and ambiguously aligned regions (Supplementary Information, S1). Out of the 12 evolutionary models, the best fitting one was determined using the Bayesian information criterion in ModelGenerator v. 0.851^58^. Phylogenetic trees were inferred using Bayesian inference and maximum likelihood, employing MrBayes v. 3.2^59^ and RAxML v. 8 software^60^, respectively. Bayesian inference trees were constructed using the Metropolis coupled Markov chain Monte Carlo algorithm with two parallel runs containing one cold and three hot chains run for 10^6^ iterations, with tree topologies sampled every 100 generations and 30% of all trees discarded as a relative burn-in period. Clade support for maximum likelihood analysis was assessed using 1,000 bootstrap replicates. The resulting phylogram was rooted using the stefin ortholog of *Brachionus plicatilis* (GenBank accession number: ANH58171) as an outgroup.

### The cloning of EnStef

Total RNA from adult *E*. *nipponicum* worms was isolated using the High Pure RNA Tissue Kit (Roche) following the manufacturer’s instructions. The transcriptor First Strand cDNA Synthesis Kit (Roche) was used for a reverse transcription of RNA to cDNA using an anchored-oligo(dT)_18_ primer. Forward (EnStefFWD: 5′-ATGCAAATGGTTGGAGGAATTGGTGCTTCA-3′) and reverse (EnStefREV: 5′-TCAAAAATATTCCAAGATATCGTCTTCAGA-3′) primers for amplification of the full-length stefin-coding sequence were designed on the basis of the 297 bp sequence of plathyhelminth stefin ortholog that was identified in the *E*. *nipponicum* transcriptome. Amplification of the cDNA ran as follows: initial denaturation (94 °C, 5 min), 35 denaturation cycles (94 °C, 1 min), primer annealing (60 °C, 1 min) and elongation (72 °C, 1 min), and a final elongation (72 °C, 10 min). The PCR products were resolved in a 1% agarose gel, purified by MinElute PCR Purification Kit (Qiagen), and cloned using the CloneJET PCR Cloning Kit (Thermo Scientific) and *E*. *coli* XL-1 blue cells (Novagen). Clones were sequenced using pJET1.2 primers from the CloneJET PCR Cloning Kit (Thermo Scientific), the BigDye Terminator v. 3.1 Ready Reaction Cycle Sequencing kit (Applied Biosystems), the BigDye X-Terminator Purification Kit, and the ABI 3130 Genetic Analyzer (Applied Biosystems). In order to verify the presence or absence of the signal peptide, 5′RACE PCR was adopted (5′/3′ RACE Kit 2nd Generation, Roche). Briefly, EnStefREV primer was used for the cDNA synthesis, while the oligo d(T)-Anchor Primer, provided by the manufacturer, and EnStefSP2 (5′-CACCAGCAACAACTTGAGTAGC-3′), a specific primer designed according to the stefin sequence, were used for the subsequent PCR. RACE PCR was performed according to the manufacturer’s instructions. PCR products were cloned and sequenced as described above. Sequences were analysed using the Geneious v. 6.1.6 software^61^.

### Expression and purification of rEnStef

The full-length EnStef gene was amplified using the rEnStefFWD (5′-CATAcata/tgATGCAAATGGTTGGAGGAATT-3′) and rEnStefREV (5′-GATAc/tcgagTCAAAAATATTCCAAGATATCGTC-3′) expression primers with NdeI/XhoI restriction sites for directional cloning (designed based on the sequence obtained after 5′ RACE PCR). PCR conditions were the same as those employed in amplification of the cDNA. Amplicons (317 bp) were digested and resolved in 1% agarose gel. After a purification by the MinElute Gel Extraction Kit (Qiagen), the digested amplicons were ligated into the corresponding cloning sites of the pET19b expression vector (Novagen). Recombinant constructs were transformed into *E*. *coli* BL21 cells (Novagen) and selected clones cultured in the Terrific Broth medium with ampicillin (100 µg/ml). Expression of rEnStef was induced at optical density 0.6 with 0.4 mM IPTG at 18 °C for 10 h. Bacterial cells were harvested by centrifugation (4,000 × g, 15 min, 4 °C). Sonication was performed on ice in 50 mM Tris buffer pH 9 with 0.5 mM EDTA and 1% Triton X-100 using a BioLogics 150 VT ultrasonic homogeniser (60% amplitude and 0.2 pulse rate for 20 min), following which the homogenised cells were centrifuged (21,000 × g, 45 min, 4 °C). The soluble fraction with highly expressed his-tagged rEnStef was separated by immobilised-metal affinity chromatography under native conditions using a nickel-charged HisTrap FF column (GE Healthcare). The purified rEnStef was then desalted on PD-10 columns (GE Healthcare). Protein purity was evaluated using SDS-PAGE under reducing conditions using Mini-PROTEAN^®^ TGX^TM^ Precast gels (4–20%, 165 V, 40 min) and a Mini-PROTEAN^®^ Tetra Cell (BIO-RAD). The gels were stained with Coomassie brilliant blue R-250 and scanned in a calibrated GS-900™ densitometer (Bio-Rad). Identification of the rEnStef in the gels was performed using mass spectrometry (see below).

### Generation of rabbit anti-rEnStef antibodies

Rabbit polyclonal antibodies against rEnStef were produced by a service laboratory of the Veterinary Research Institute (Brno, Czech Republic). Rabbits were primed by a subcutaneous injection using 500 µg of the antigen emulsified with a complete Freund’s adjuvant (50%) and boosted by the same amount of antigen in a 50% incomplete Freund’s adjuvant three weeks later. Immune sera were sampled four weeks after the initial injection of the antigen. Pre-immune sera collected before immunisation were used as negative controls.

### Western blot detection of EnStef in the excretory-secretory products and soluble worm extract

The presence of a native stefin in excretory-secretory products and in the soluble worm extract as well as reactivity of antibodies with rEnStef were verified on blots of samples resolved by SDS-PAGE under the conditions described above. Protein bands were transferred onto the PVDF membrane (Bio-Rad). After blocking (10 mM PBS, pH 7.2, containing 5% non-fat milk, 2.5% BSA and 0.05% Tween 20) for 1 h, the membranes were incubated overnight with a rabbit anti-rEnStef or control sera (1:50) in the blocking buffer. After washing in PBS (3 × 3 min), the membranes were incubated in a peroxidase-conjugated goat anti-rabbit IgG (Sigma; 1:400) in the blocking buffer for 2 h. Reactions were visualised by the Opti-4CN^TM^ substrate kit (Bio-Rad) used according to manufacturer’s instructions.

### Mass spectrometry analysis

The presence of rEnStef in the bacterial lysate and of a native EnStef in excretory-secretory products and soluble worm extract were verified by mass spectrometry. Proteins were reduced (DTT) and alkylated (iodoacetic acid) before being incubated with trypsin (sequencing grade; Promega) for 2 h at 40 °C. Tryptic peptides were extracted into LC-MS vials^62^ and concentrated in a SpeedVac concentrator (Thermo Fisher Scientific).

LC-MS/MS analyses were undertaken using a RSLCnano system connected to an Orbitrap Elite hybrid spectrometer (Thermo Fisher Scientific). Tryptic digests were concentrated online and desalted using a trapping column (100 μm × 30 mm; 3.5-μm X-Bridge BEH 130 C18 sorbent – Waters). Then they were separated on an Acclaim Pepmap100 C18 analytical column (3 µm particles, 75 μm × 500 mm; Thermo Fisher Scientific) using the 50 min (in-gel digests) and 100 min (peptide mixtures from FASP) non-linear gradient program of mobile phase A (0.1% FA in water) and mobile phase B (0.1% FA in 80% acetonitrile). The analytical column outlet was directly connected to a Digital PicoView 550 (PicoTip emitter SilicaTip; New Objective) with ABIRD (Active Background Ion Reduction Device) installed. MS data were acquired using a data-dependent strategy selecting up to 10 of the top precursors for the HCD MS/MS spectra acquisition.

Analysis of the mass spectrometric RAW data files was carried out using Proteome Discoverer software v. 1.4 (Thermo Fisher Scientific) with the in-house Mascot v. 2.5.1 (Matrixscience, London, UK) search engine. MS/MS ion searches were first undertaken against the modified cRAP database (http://www.thegpm.org/crap/), whereby the spectra unassigned (Mascot ion score < 30) during the first search were submitted to the main database search against the in-house protein database for *E*. *nipponicum* containing the EnStef protein (derived from nucleotide sequence data; E_nip_trans_39617_m.270033) concatenated with NCBI database for *C*. *carpio* (version from 12.1.2017; downloaded from https://www.ncbi.nlm.nih.gov/protein/?term=txid7962%5bOrganism:exp%5d). The concatenated database contained a total of 142,294 sequences. Mass tolerance for peptides and MS/MS fragments was 7 ppm and 0.03 Da, respectively. Two enzyme (trypsin) miss cleavages as well as oxidation of methionine, deamidation (N, Q), propionamidation (C), and acetylation (protein N-terminus) were used as optional modifications and set as parameters for all searches. Database search results were evaluated using the Mascot ion score (significant identity or extensive homology p < 0.05; score > 28) and only rank 1 spectrum matches and peptides at least 6 amino acids long were taken into consideration.

### Inhibition assays

Recombinant forms of *E*. *nipponicum* cathepsin B (EnCB, 10 nM) and cathepsin L3 (EnCL3, 10 nM), previously produced by the co-authors (HD and LJ, awaiting publication), as well as recombinant mouse cathepsin L (MmCL, 5 mM; R&D Systems) and recombinant *Ixodes ricinus* asparaginyl endopeptidase (IrAe, 10 nM), kindly provided by Daniel Sojka (Institute of Parasitology, Czech Academy of Sciences, České Budějovice) were used to test the inhibitory effect of rEnStef at increasing concentrations in a continuous fluorometric assay. EnCL3 and MmCL were first activated in 50 mM sodium citrate, 150 mM NaCl, 1 mM EDTA, and 0.615% CHAPS, pH 3.0 for 10 min. IrAe was activated as previously described^63^. All reactions were performed in 25 mM MES buffer pH 6 containing 5 mM DTT. Preincubation of rEnStef (10–1,000 nmol) with each peptidase in the assay buffer was performed in 96-well black, flat-bottomed plates for 10 min at room temperature. Subsequently, 10 µM peptidase-specific fluorogenic peptidyl substrate was added (Z-Phe-Arg-AMC for EnCB, EnCL3 and MmCL, and Z-Ala-Ala-Asn-AMC for IrAe) and the relative fluorescence of released aminomethylcoumarin measured at 1 min intervals in a SpectraMax i3 fluorometer (Molecular Devices) at excitation and emission wavelengths 355 and 460 nm, respectively. The data were normalised between 0 and 100% activity, and the IC_50_ values were calculated by non-linear regression using GraphPad Prism 7 software (San Diego, CA, USA).

### Inhibition of haemoglobinolysis by rEnStef

The inhibitory effect of rEnStef on a proteolytic cleavage of haemoglobin was tested in the presence of *E*. *nipponicum*-derived excretory-secretory products (3 µg), soluble worm extract (1.5 µg), and recombinant EnCL3 (1 µg). Each of the samples was preincubated with 3 µg of rEnStef in 0.1 M phosphate-citrate buffer pH 5 with 2 mM DTT for 10 min at room temperature. Bovine haemoglobin (1 µg; Sigma) was added to the samples, which were then incubated for 16 h at 30 °C. Controls were represented by either a peptidase-containing material with haemoglobin, or by haemoglobin alone. All samples were resolved by SDS-PAGE as described above.

Data sets generated during and/or analysed during the current study are available from the corresponding author upon reasonable request.

### Data availability

#### Accession Codes

The EnStef sequence was deposited in the NCBI GenBank under accession number KY192529. *E*. *nipponicum* Transcriptome Shotgun Assembly project has been deposited at DDBJ/EMBL/GenBank under the accession GFYM00000000. EnStef sequence in the transcriptome is accessible under E_nip_trans_39617_m.270033.

## Electronic supplementary material

Supplementary Information
