# Supplementary material for: A novel type I cystatin of parasite origin with atypical legumain-binding domain
Source: Sci Rep. 2017 Dec 13;7:17526. doi: 10.1038/s41598-017-17598-2 (PMC5727476; doi:10.1038/s41598-017-17598-2)
Supplement: Supplementary file 1 — Supplementary Information [file 41598_2017_17598_MOESM1_ESM.pdf]

A novel type I cystatin of parasite origin with atypical legumain-binding domain

Jana Ilgová[1*], Lucie Jedličková[2], Hana Dvořáková[2], Michal Benovics[1], Libor Mikeš [2], Lubomír Janda[3], Jiří Vorel[1], Pavel Roudnický[1], David Potěšil[3], Zbyněk Zdráhal[3], Milan Gelnar[1] and Martin Kašný[1,2]

Supplementary Information

S1. Multiple protein sequence alignment used for the phylogenetic reconstruction of platyhelminth stefin orthologs.

```
                        1       10        20        30        40        50        60
                        |        |         |         |         |         |         |
E. nipponicum           MQM-VGGIGASRTPTDEERSKLHGVIAAHLQSNSLPHEP--LSLLSIATQVVAGVNYFVK
O. viverrini            MPL-CGGVGDARTPTPEEKQKLESVLHQSLHTH-LGSKPDSLEVVQVATQVVSGTNYFAK
C. sinensis             MPI-CGGISAARIPTADEKKKLEPVLLQSLYAH-LGSKPTSAEVVLVATQVVAGTNYFAK
S. japonicum            MPLCCGGVGAPREPSVEEKQKLKTLLENNLEAH-IGRKPPVCDIVQVSSQVVAGTNYFVK
S. haematobium          MPLCCGGIGTPREPSAEEKEKLKTLLESKLESH-IGRKPRSFEIVQITSQVVAGTNHFVK
S. mansoni              MPLCCGGIGTPREPSAEEKEKLKTLLESKLESH-IGRKPRSFEIVQITSQVVAGTNHFVK
F. gigantica            M-M-CGGCTQTRAPSAEEKTELETVLREQLESH-IGHKPQIIEVVEICTQVVAGINYFAK
S. erinaceieuropaei     MMM-CGGASSCRAPSEEEKRLLLPPLSAHLEGR-LGKSPQDVEIIEVRTQTVAGTNYFLK
T. solium               MPM-CGGLSASVQPSDEDKQRLTPVVKDYIAQQ-TGQEPSEVKITEVSRQLVNGINHFLK
T. saginata             MPM-CGGLSASVQPSDEDKQRFTPVVKDYITQQ-TGQEPSEVKITEVSRQLVNGINHFLK
T. asiatica             MPM-CGGLSASVQPSDEDKQRLTPVVKDYITQQ-TGQEPSEVKIAEVSRQLVNGINHFLK
E. granulosus           MPM-CGGLTTSVRPSDEDKQLLTPVIKDYIAQQ-LGQEPSEVKITEVSRQIVNGTNHFLK
E. multilocularis       MPM-CGGLTTSVRPSNEDKQLLTPVVKDYIAQQ-LGREPSEVKITEVSRQIVNGTNHFLK
G. salaris              MIK-CGGTSEAREASQEEIDLLLPVISKYMVD--VKLDNKSLEIVHIKTQVVAGTMYYVR
B. plicatilis           M-M-VGGLGQAQ-PANEEIQGLVNQVKPQLATHAPGHESKDLKAVSFRSQVVAGTNYFIK

                        61      70        80        90        100
                        |        |         |         |         |
E. nipponicum           VKH-GDNVSHYRIYEKLPCYGSTIEVSSVLHGKSEDDILEYF
O. viverrini            VKLNGDNYVHARIYEKLPCHGGTTELHSIQKDKTHADPLGYF
C. sinensis             VKVNNDHYIHTRVYEQLPCYGGALELHSVQMNKTDTDPLDYF
S. japonicum            VHVGDDEYVHARIFEPLPCHGKELQLHSVLKDKKKNDALEYF
S. haematobium          VKIDDGDYIHARIFEPLPCHGKELQLHSVVKDKKESDALEYF
S. mansoni              VKLDNGEYIHARIFEPLPCHGKELQLHSVVKDKKESDALEYF
F. gigantica            VKIGNDNYIHARIFQDLPCNGGKKKVHSVLKDKSATDSLKYF
S. erinaceieuropaei     VKHSDGKVCHVRVFKALPCNGGDIEVVKVVEKSGLGDPLEYF
T. solium               VEH-GDNVWHIRVHEALPCYGSKIEVHS-HKVAAAGDPLTYF
T. saginata             VEH-GGNVWHIRVHEALPCYGSKIEVHS-HKVAAAGDPLTYF
T. asiatica             VEH-GGSVWHIRVHEALPCYGSKIEVHS-HKVAAAGDPLTYF
E. granulosus           VEH-DGNCWHVRVHEALPCYGGKVEVHS-HKVASVGDPLTYF
E. multilocularis       VEH-DGNCWHVRVHEALPCYGGKVEVHS-HKVASVGDPLTYF
G. salaris              VKH-GETFTHFKIMKKLPCHGSEMSVMN-HKHAGEEKEVQYF
B. plicatilis           VHA-GDQHLHLKVHKPLPHTGNPPELSGVQAGKSHEDEVAFF
```
